# Medical Care Disruptions During the First Six-Months of the COVID19 Pandemic: The Experience of Older Breast Cancer Survivors

**DOI:** 10.21203/rs.3.rs-416077/v1

**Published:** 2021-04-14

**Authors:** A Dilawari, KE Rentscher, W Zhai, X Zhou, TA Ahles, J Ahn, TN Bethea, JE Carroll, HJ Cohen, DA Graham, HSL Jim, B McDonald, ZM Nakamura, SK Patel, JC Root, BJ Small, AJ Saykin, D Tometich, K Van Dyk, JS Mandelblatt, Thinking and Living with Cancer Study

**Affiliations:** 1 Medstar Washington Hospital Center Washington, DC; 2 Lombardi Comprehensive Cancer Center, Georgetown University, Washington, DC; 3 Cousins Center for Psychoneuroimmunology, University of California, Los Angeles, Los Angeles, CA; 4 Semel Institute for Neuroscience and Human Behavior, Department of Psychiatry & Biobehavioral Sciences, University of California, Los Angeles, Los Angeles, CA; 5 Department of Biostatistics, Bioinformatics, and Biomathematics, Georgetown University, Washington, DC; 6 Memorial Sloan Kettering Cancer Center, New York, NY; 7 Center for the Study of Aging and Human Development, Duke University Medical Center, Durham, NC; 8 John Theurer Cancer Center, Hackensack University Medical Center, Hackensack, NJ; 9 Moffitt Cancer Center, Tampa, FL; 10 Department of Radiology and Imaging Sciences, Indiana University School of Medicine and Indiana University Melvin and Bren Simon Cancer Center, Indianapolis, IN; 11 Department of Psychiatry, University of North Carolina–Chapel Hill, Chapel Hill, NC; 12 City of Hope National Medical Center, Los Angeles, CA; 13 University of South Florida, Tampa, FL

**Keywords:** medical care disruptions, COVID, cancer survivors, breast cancer, older adults

## Abstract

***Purpose*.:**

Older cancer survivors required medical care during the COVID-19 pandemic despite infection risks, but there are limited data on medical care in this age group.

**Methods.:**

We evaluated care disruptions in a longitudinal cohort of non-metastatic breast cancer survivors ages 60–98 from five US regions (n=321). Survivors completed a web-based or telephone survey from May 27, 2020 to September 11, 2020. Care disruptions included self-reported interruptions in ability to see doctors, receive treatment or supportive therapies, or fill prescriptions. Logistic regression models evaluated bivariate and multivariate associations between care disruptions and education, medical, psychosocial and COVID-19-related factors. Multivariate models included age, county COVID-19 rates, comorbidity and post-diagnosis time.

***Results*.:**

There was a high response rate (n=262, 81.6%). Survivors were 32.2 months post-diagnosis (SD 17.5, range 4–73). Nearly half (48%) reported a medical disruption. The unadjusted odds of care disruptions were significantly higher with more education (OR 1.23 per one-year increase, 95% CI 1.09–1.39, p =0.001) and greater depression (OR 1.04 per one-point increase in CES-D score, CI 1.003–1.08, p=0.033); tangible support decreased the odds of disruptions (OR 0.99, 95% CI 0.97–0.99 per one-point increase, p=0.012). There was a trend for associations between disruptions and comorbidity (unadjusted OR 1.13 per 1 added comorbidity, 95% CI 0.99–1.29, p=0.07). Adjusting for covariates, only higher education (p=0.001) and tangible social support (p=0.006) remained significantly associated with having care disruptions.

**Conclusions.:**

Older breast cancer survivors reported high rates of medical care disruptions during the COVID-19 pandemic and psychosocial factors were associated with care disruptions.

## Introduction

The COVID-19 pandemic has had broad effects on medical care delivery, with delays in routine care and postponement of non-COVID-19 related services [1][2]. The risks of delaying routine care during the COVID-19 pandemic have been high among those with chronic illnesses, including cancer [3]. These same groups also have the highest risk of severe complications and mortality from COVID-19 infection. Cancer care guidelines during the pandemic have focused on treatment for new patients [4][5]. There has been less attention to the impact of the pandemic on existing cancer survivors or associated disruptions in care. A proportion of long-term cancer survivors are in older age groups and may be especially vulnerable to disruptions that could adversely affect survivorship care [6]. Additionally, pandemic-related social isolation could exacerbate depression, anxiety and sleep disturbances after cancer, increasing the need for medical care [7][8].

In this study, we describe the impact of the COVID-19 pandemic on the medical care of older breast cancer survivors enrolled in the Thinking and Living with Cancer (TLC) longitudinal cohort study. We describe the prevalence of care disruptions and explore factors that might be associated with disruptions that occurred during the first six-months of the pandemic. The results are intended to inform survivorship care during and beyond the pandemic.

## Methods

TLC is an Institutional Review Board approved study that has been reported previously [9] and was conducted across sites in five regions . The COVID survey used in this study was IRB approved at all sites.

### Population

The target population included 705 survivors recruited between August 1, 2010 and March 1, 2020 that were 60 years or older and newly diagnosed with AJCC stage 0-III breast cancer at study entry. Those with neurological disorders or hearing or vision impairments that precluded assessment, had a history of other cancers or any prior chemotherapy, or were non-English-speaking were excluded.

**Figure 1:**
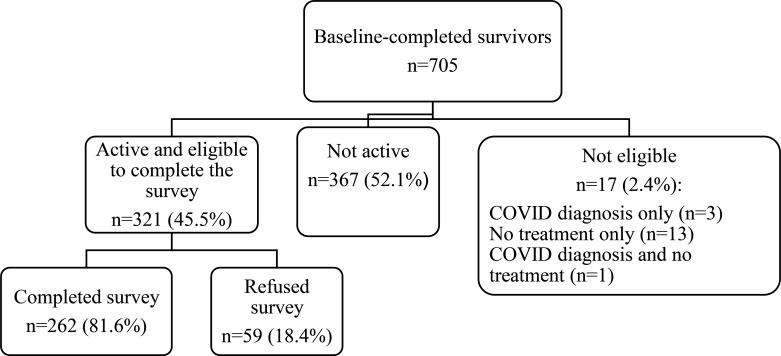
A sample for evaluation of medical disruptions in older breast cancer survivors . Participants were excluded if they were not active (i.e. had completed the study, dropped out, or deceased) since the start of survey data collection. The percentage who completed and refused was calculated among those active and eligible to complete the survey. Eligibility for completing the survey included no COVID-19 diagnosis and receiving treatment.

For the current study, we excluded survivors were no longer active in the study, including those that had had a recurrence, had completed all study follow-up, dropped out of the study, or died (n=367). We also excluded survivors with missing treatment data or who had been diagnosed with COVID-19 (n=17). Among the 321 women eligible for this study, 81.6% (n=262) completed the COVID-19 survey and constitute the analytic sample (Figure 1). The survivors completing the survey were similar to the non-completers, except they were more likely to be White (84.7% vs. 72.9%, respectively, p=.03). The analytic sample was also similar to the overall target population, except for having slightly more comorbidities (3.0 [SD 2.1] vs 2.5 [SD 1.9], p<.01) and a lower rate of chemotherapy receipt (20.6% vs 29.7%, p<.01).

### Data Collection

As a part of TLC assessments survivors has completed a baseline, pre-systemic therapy survey at enrollment with annual follow-up. The COVID survey was developed and reviewed by a committee of TLC investigators and included standard study assessments, items from the National Institute of Mental Health (NIMH) Psychosocial Impact of COVID-19 survey [10], and additional COVID-related measures. The survey was conducted online between May 27, 2020-September 11, 2020. Participants who did not have an email address on file were called to provide one. If participants did not respond to the initial emailed invitation, the link was automatically re-sent every week for 3 weeks; if no response, study staff called the participant to complete the survey by phone. Most completed the survey online (87.2%).

### Measures

The outcome measure was having any medical care disruption during the pandemic (yes/no) based on response to items adapted from the NIMH-NIH survey [10]: ability to see doctors, receive medical treatment, fill prescriptions, or receive supportive therapies (e.g. physical therapy, massage, acupuncture).

We examined potential factors hypothesized to be associated with care disruptions. Pandemic-specific factors included cumulative per capita rates of COVID-19 deaths in the county of the participants’ residence through the week of survey completion [11][12]. Pandemic-related worry was assessed based on items from the NIMH-NIH survey [10]: job loss (self or family), loss of insurance, and worries about being infected with COVID-19, food access, financial issues, housing, and transportation during the pandemic; each item was rated on a 1–10 scale from not worried to very worried.

Socio-demographic factors include age, race (White vs, non-White) and years of education. Clinical factors included AJCC cancer stage, time from diagnosis and type of systemic therapy at enrollment and number of comorbidities on the last pre-COVID assessment. Psychosocial variables from the last pre-COVID assessment included: anxiety (20-item State Trait Anxiety Inventory [STAI])[13], depression (20-item Center for Epidemiologic Studies–Depression [CES-D] Scale, [14] two questions from the CES-D about sleep disturbance, emotional and tangible support subscales of the MOS [15], and quality of life (Functional Assessment of Cancer Therapy- General [FACT-G]) [16].

### Statistical Analysis

Univariate logistic regression methods were used as a first step to describe the association between having medical care disruption vs. not and covariates. Variables with p<0.1 association in the univariate logistic regression were then considered in a multivariable logistic regression model. Age, education, time from diagnosis, cumulative per capita rate of deaths, and comorbidities were also retained in the final model for face validity. We determined the final multivariable model using backward selection with a threshold of p<0.1. Goodness-of-fit was reported based on Akaike Information criterion (AIC), Bayesian Information Criterion (BIC), and the concordance statistics (C-statistics). Odds ratios and corresponding 95% confidence intervals were provided for all analyses. Statistical significance was determined with a two-sided p-value <0.05. All analyses were conducted in SAS Version 9.4.b (SAS Institute Inc., Cary, NC, USA).

## Results

The survivors’ average age was 68 years (range 60–82) and 97.7% had internet access. The majority (66.4%) were ≥2 years from breast cancer diagnosis with 22.8% diagnosed within the preceding year (Table 1). Nearly one-half (48%) of survivors reported having had any medical disruption during the first six months of the pandemic. Disruptions included interruptions in seeing or speaking to their doctor (reported by 48.4%), disruptions in medical treatments (51.2%), and difficulty obtaining supportive care therapies (40.2%). While the mean number of medications was 3.8 (SD 2.2), only 4.7% of survivors reported difficulty filling prescriptions.

Several factors were associated with having medical care disruptions in bivariate analyses (Table 1). For each additional comorbidity, there was an increase in reporting medical disruptions (OR 1.13, 95% CI 0.99–1.29, p=0.071). More years of education was significantly associated with the odds of reporting medical care disruptions (OR 1.23 per one year increase, 95% CI 1.09–1.39, p =0.001). More tangible support pre-COVID was associated with not having medical disruptions during the pandemic (p=0.012). Survivors with higher CES-D scores (more depressive symptoms) at their last pre-COVID assessment were also more likely to report medical disruptions (OR 1.04 per one-point increase in depressive symptoms, 95% CI 1.003–1.080, p=0.033). Participants with better quality of life pre-COVID were less likely to experience medical disruptions (OR 0.95 per one point increase in FACT-G score, 95% CI 0.932–0.980, p<0.001). There were no significant associations of care disruptions with cancer-specific factors such as time since diagnosis, stage or initial therapy.

In multivariable-adjusted analyses, only tangible social support and education remained significantly associated with medical disruptions: the odds of reporting disruptions were lower for those with more tangible social support pre-COVID (OR 0.98 per 1-point per increase, 95% CI 0.97–0.995, p=0.006) and 23% higher for each increase in years of education (OR 1.23, 95% CI 1.09–1.39, p=0.001) (Table 1).

## Discussion

This study examined health care access among older US breast cancer survivors in the first six-months of the COVID-19 pandemic. Nearly one-half of these older survivors reported experiencing medical care disruptions. Having more tangible social support reduced the odds of having medical care disruptions during the pandemic. Survivors with more education reported more disruptions than those with less education though the mean number of years of education for the participants was very high.

About half the survivors reported difficulties seeing or speaking with their doctors or receiving supportive therapies (including integrative treatments and physical therapy). The latter finding may reflect some institutions’ protocols prioritizing medical therapies rather than supportive care during the pandemic [1][3][17]. Since these older cancer survivors are part of a longitudinal cohort study, we may be able to assess the impact of care disruptions on subsequent quality of life in the future.

The rate of medical care disruptions among older breast cancer survivors that we observed was consistent with rates reported from general populations. The Centers for Disease Control (CDC) reported that approximately 41% of U.S. adults delayed or avoided routine and urgent medical care due to the COVID-19 pandemic [18] and studies worldwide similarly indicated a rise in missed medical appointments during the pandemic [19][20]. Most studies that included cancer patients or survivors have focused on newly diagnosed and younger patients[5], with limited information on disruptions experienced by long-term or older cancer survivors [18–2][21]. Our cohort was on average, two to three years from diagnosis and might be less vulnerable to care disruptions as women newly diagnosed with breast cancer. However, older survivors have more comorbidities than younger patients so care disruptions could have a larger impact on health.

The results of this study also highlight how common issues affecting cancer survivors such as social support can buffer disruptions in medical care during the pandemic. This observation may reflect the direct impact of social support on transportation to medical care or arranging appointments. Alternatively, social support may be capturing other aspects of cancer survivors’ lives not captured by our measures, including having more social connections. The positive effects of social support in cancer survivorship have been reported in other studies. The Nurses’ Health Study demonstrated that the degree of social support in breast cancer survivors affected physical function and adverse cancer-related symptoms [22], and other studies have shown associations between social isolation and increased mortality for cancer survivors [23]. However, there is less literature on the impact of social support specifically on obtaining medical care.

Contrary to expectation we found that as years of education increased the odds of reporting medical care disruptions increased. This may be related to higher education being associated with more general awareness of the need for health maintenance, as lower education and health literacy has been linked to less use of screening and routine preventive care appointments [24]. Alternatively, higher education may be a proxy for a greater desire for medical care or increased caution about COVID risk. It will be important to compare our findings to newer reports on health care disruptions.

The study has many strengths including the ability to consider COVID-related medical care disruptions in the context of an ongoing longitudinal cohort of breast cancer survivorship, focusing on older cancer survivors, and having data on preexisting factors that could affect survivors’ ability to respond to unexpected events. However, there are limitations that should be considered in evaluating our results. First, fewer non-White survivors in the cohort responded to the survey than White participants. Non-White adults have had higher infection rates and greater economic losses than White adults during the pandemic [25]. To the extent that older non-White survivors were under-represented, we could have under-estimated the overall rate of medical care disruptions among breast cancer survivors. Second, our cohort was highly educated and the majority had health insurance through Medicare, so their experiences may not reflect access to health care among other survivor groups. Third, while we considered the per capita regional rate of COVID-19 deaths, there are variations in effects of state or local lockdown orders, social distancing, and media messages that we did not capture in our measures.

Overall, this study shows that during the first six months of the COVID-19 pandemic nearly 50% of older breast cancer survivors experienced some type of disruption in medical care. It will be important to determine if these disruptions persist or resolve as a larger proportion of the population becomes vaccinated or whether the initial disruptions in care will have long-lasting effects on health and function. Until then, older breast cancer survivors appear vulnerable to losses in medical care and should be considered in future studies of the growing impact of the COVID-19 pandemic on health care.

## Figures and Tables

**Table 1:** Odds of Having Medical Care Disruptions During the COVID19 Pandemic Among Older Breast Cancer Survivors

	No Disruptions N=135	Disruptions N=127	Factors Associated with Having Medical Care Disruptions			
	Percent (n) or mean (SD)		Unadjusted OR (95% CI)	P value	Adjusted OR (95% CI)^1^	P value
**Demographic factors**						
Age, years	68.0 (5.2)	67.8 (5.5)	0.993 (0.95,1.04)	0.753	0.99 (0.95,1.04)	0.548
Race					-----	
Non-White	47.5 (19)	52.5 (21)	1.21 (0.62,2.37)	0.580		
White	52.3 (116)	47.7 (106)	Reference			
Education, years	15.1 (2.3)	16.0 (2.0)	1.22 (1.08,1.37)	<.001	1.23 (1.09,1.39)	0.001
**Clinical factors**						
Months from diagnosis	33.5 (18.0)	30.6 (16.7)	0.99 (0.98,1.01)	0.191	0.99 (0.97,1.01)	0.168
Stage (AJCC v.6)						
0-I	52.2 (108)	47.8 (99)	referent		-----	
II or III	48.9 (22)	51.1 (23)	1.14 (0.60,2.17)	0.689		
Systemic Treatment					-----	
Chemotherapy +/− hormonal	45.1 (23)	54.9 (28)	1.38 (0.75,2.55)	0.307		
Hormonal only	53.1 (112)	46.9 (99)	referent			
Comorbidities prior to COVID, number	2.7 (1.8)	3.1 (2.0)	1.13 (0.99,1.29)	0.071	1.09 (0.94,1.26)	0.238
Prescription drugs prior to COVID, number	3.6 (2.2)	4.0 (2.6)	1.08 (0.97,1.19)	0.170	-----	
**COVID-related factors**						
Family/household member lost their job	34.5 (10)	65.5 (19)	2.20 (0.98,4.93)	0.056	-----	
COVID-related worries, per 1-point increase^2^	16.8(6.5)	18.5(9.2)	1.03 (1.00,1.06)	0.085	-----	
Per capita COVID deaths in county per 1000^3^	0.9 (0.9)	0.8 (0.8)	0.86 (0.65,1.15)	0.319	0.80 (0.58,1.11)	0.179
**Psychosocial factors prior to COVID**						
Depressive symptoms ^4^	5.5 (5.4)	7.5 (8.6)	1.04 (1.00,1.08)	0.033	-----	
Anxiety^5^	27.5 (5.5)	28.8 (7.5)	1.03 (0.99,1.07)	0.120	-----	
Tangible social support, per one point increase^6^	83.8 (19.3)	76.9 (23.8)	0.99 (0.97,1.00)	0.012	0.98 (0.97,1.00)	0.006
Emotional support per one point increase^7^	82.7 (17.9)	78.1 (20.4)	0.987 (0.98,1.00)	0.053	-----	
Sleep disturbance^7^	52.4% (44)	47.6 (40)	0.95 (0.57,1.60)	0.849	-----	
FACT– G Total, per 1 point increase ^8^	71.0 (9.1)	66.2 (11.8)	0.96 (0.93,0.98)	<.001	-----	
Model fit statistics				BIC=365.11; AIC=340.37; C=0.673		

1. Logistic regression including all variables on the table.

2 COVID-related worries based on 7-items from COVID survey. Scores range from 7–70, with higher scores reflecting more covid worries.

3 Based on cumulative death rates per capita in county of residence from pandemic to date of interview per 1000 based on data reported to the NY Times [11] and the US Census [12].

4 Based on the CES-D, Center for Epidemiologic Studies Depression Scale [**Error! Bookmark not defined.**]. Scores range from 0–60, with higher scores reflecting more psychological distress.

5 Based on the State-Trait Anxiety Inventory [13]. Scores range from 20 to 80, with higher scores reflecting more anxiety.

6 Based on the normalized MOS-Tangible social support [15]. Scores range from 0 to 100, with higher scores reflecting more tangible social support.

7 The presence of a sleep disturbance (yes/no) was determined from the endorsement of one or both of 2 questions [25] from CES-D: During the last 7 days, I have been sleeping well” (with subjects who reported “not at all” or “a little bit” coded as having a sleep disturbance) and “During the past week, my sleep was restless” (with subjects who reported “occasionally or moderate amount of time” or “most or all the time” coded as having a sleep disturbance.

8 Based on the FACT-G [16]. Scores range from 0 to 84, with higher scores reflecting better functioning.
